# Recent Advances on Internet of Things

**DOI:** 10.1155/2014/709345

**Published:** 2014-12-22

**Authors:** Xiaoxuan Meng, Jaime Lloret, Xudong Zhu, Zhongmei Zhou

**Affiliations:** ^1^VMware, 3401 Hillview Avenue, Palo Alto, CA 94304, USA; ^2^Department of Communications, Universidad Politecnica de Valencia, Spain; ^3^Department of Computer Science and Engineering, Minnan Normal University, Zhangzhou, China; ^4^School of Information and Electronic Engineering, Zhejiang Gongshang University, Hangzhou 310018, China

Recently, internet of things (IoT) has become increasingly ubiquitous. IoT enables a detailed characterization of the physical environment, as well as a rich set of interactions with the physical world. Therefore, IoT has the potential to revolutionize pervasive computing and its applications. The success of intelligent IoT highly depends on the system architectures, networks and communications, data processing, and ubiquitous computing technologies, which support efficient and reliable physical and cyber interconnections. Indeed, the realization of a ubiquitous IoT poses several challenges about seamless integration, heterogeneity, scalability, mobility, and many others.

In this special issue, we mainly focus on the latest advancements of IoT. We invite scientists and investigators to contribute to this special issue with original research articles and review articles on theories and key technologies for scientific and engineering problems in IoT, as well as their applications to conquer engineering problems.

Generally, the accepted papers in this special issue can be divided into several categories. Most of the papers focus on the key technologies for IoT, like wireless sensor networks (WSNs), video and image processing, distributed systems, and so forth. There are also papers related to algorithms and model for IoT, for example, bee colony algorithm, parallelized dilate algorithm, color gamut description algorithm, and so forth. The rest of the papers are talking about the applications of IoT in real life.

In all, we hope that readers will find in this special issue not only the new ideas, cutting-edge information, new technologies, and applications of IoT but also a special emphasis on how to solve various emerging problems by using new technologies and algorithms.

*Xiaoxuan Meng* *Xiaoxuan Meng* *Jaime Lloret* *Jaime Lloret* *Xudong Zhu* *Xudong Zhu* *Zhongmei Zhou* *Zhongmei Zhou*

